# Immunotherapy and local therapies in metastatic laryngeal cancer management: a case report

**DOI:** 10.3332/ecancer.2025.1947

**Published:** 2025-07-17

**Authors:** João Felipe Lima Feldmann, João Henrique Lima Feldmann, Cassio Murilo Hidalgo-Filho, Amanda Acioli de Almeida Robatto, Breno Jeha Araújo, Publio Cesar Cavalcante Viana, Gilberto de Castro Junior

**Affiliations:** 1 Clinical Oncology Department, Hospital Sírio-Libanês, São Paulo, SP 01308-050, Brazil; 2 Radiology Department, Hospital Sírio-Libanês, São Paulo, SP 01308-050, Brazil; a https://orcid.org/0009-0005-6435-1349

**Keywords:** laryngeal carcinoma, immunotherapy, local therapy, Oligometastasis

## Abstract

**Background:**

Advanced laryngeal carcinoma (LC) has a poor prognosis with limited treatment options. Managing oligometastasis is challenging, and there are currently no standard recommendations.

**Methods:**

We reported a case of a 64-year-old male with locally advanced LC who developed oligometastatic disease in the bones and liver 21 months after concurrent cisplatin-based chemoradiotherapy. Initially, due to negative PD-L1 expression, the patient was treated docetaxel, cisplatin and cetuximab combination. Chemotherapy after 10 months, new hepatic progression was confirmed by biopsy. Given the asymptomatic, single-site progression in a cirrhotic liver, microwave ablation was performed. Isolated bone progressions were treated with stereotactic body radiation therapy at 2 and 4 months, and nivolumab replaced cetuximab.

**Results:**

The patient has shown no evidence of disease progression for 22 months, with excellent tolerance.

**Conclusion:**

The synergy between nivolumab and local therapies appears promising for managing oligometastasis in laryngeal cancer.

## Background

Laryngeal carcinoma (LC) is second in prevalence among malignant neoplasms affecting the upper aerodigestive tract. According to data from GLOBOCAN 2022, there were 189,191 new cases and 103,359 deaths, primarily impacting men in their sixth and seventh decades who are chronic smokers [1]. Early-stage LC typically exhibits favourable responses to monotherapy, such as surgery or radiotherapy, while advanced cases necessitate multimodal interventions. Despite advances in treatment modalities, 5-year survival rates for supraglottic LC stand at 45%; however, when considering the subgroup with metastatic spread, which constitutes 15%–20% of cases, these rates decline alarmingly to 30%, as reported by the American Cancer Society [2]. Among these patients, 40% present with oligometastatic disease. This condition, characterised by one to five metastases, remains potentially amenable to curative treatments and may benefit significantly from local therapies [3].

Local therapies offer benefits such as reduced morbidity and the potential for outpatient treatment. Currently, advanced disease is often treated with polychemotherapy, with or without anti-EGFR monoclonal antibodies like cetuximab. However, this approach frequently leads to suboptimal oncological outcomes at the expense of high toxicity. More recently, the KEYNOTE-048 trial has shown that adding pembrolizumab to first-line chemotherapy improves efficacy safely. This combination appears more effective with higher PD-L1 expression [4]. In second-line treatment, nivolumab has improved overall survival according to CheckMate 141 data, regardless of PD-L1 expression, underscoring the potential benefit of immunotherapy in contexts where standard cytotoxic treatments are ineffective [5]. Combining immunotherapy with local therapies holds promise due to its theoretical ability to induce a significant release of neoantigens into circulation and promote immunomodulation of the tumour microenvironment, rendering it more receptive to immunotherapy. Given the magnitude and duration of the clinical response to immunotherapy observed in a highly unlikely clinical context, we present this case.

## Case report

A 64-year-old male patient, an active smoker with a 66-pack-year history, who also has a history of alcohol misuse and was previously diagnosed with systemic arterial hypertension, dyslipidemia, hyperthyroidism and liver cirrhosis, presented with laryngeal irritation in early 2019. Due to the persistence of symptoms, the patient underwent a neck computed tomography (CT) scan and laryngoscopy, revealing an ulcer-infiltrative lesion of the epiglottis with extension to adjacent structures. A biopsy of the epiglottic lesion discovered an invasive keratinising squamous cell carcinoma of the supraglottic region. Fluorine-18 fluorodeoxyglucose-labeled positron emission tomography/computed tomography (whole-body FDG PET/CT) showed no evidence of distant metastatic disease. According to the 8th edition of the American Joint Committee on Cancer (AJCC), the tumor was staged as stage IVA laryngeal cancer based on the TNM (Tumor, Node, Metastasis) classification (T3N2bM0). A concurrent cisplatin-based radiotherapy and chemotherapy protocol was administered between September and November 2019 to preserve the larynx, resulting in a complete clinical response.

**Figure 1.**
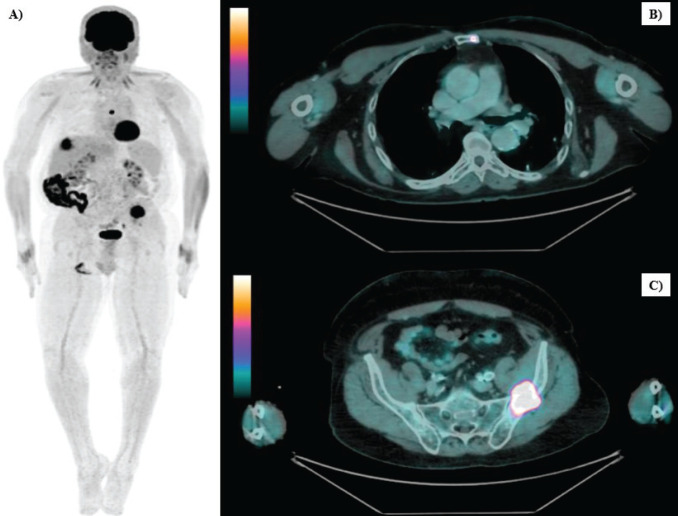
Whole-body FDG PET/CT images of secondary bone implants. (a): Whole-body maximum intensity projection (MIP) image showing abnormal FDG uptake in the left iliac bone wing, sternal region and a nodule in segment VII of the liver. (b): Sagittal view of a lytic bone lesion in the left side of the sternal body (SUVmax: 17.4). (c): Sagittal view of a metastatic implant in the left iliac bone (SUVmax: 18.5).

**Figure 2.**
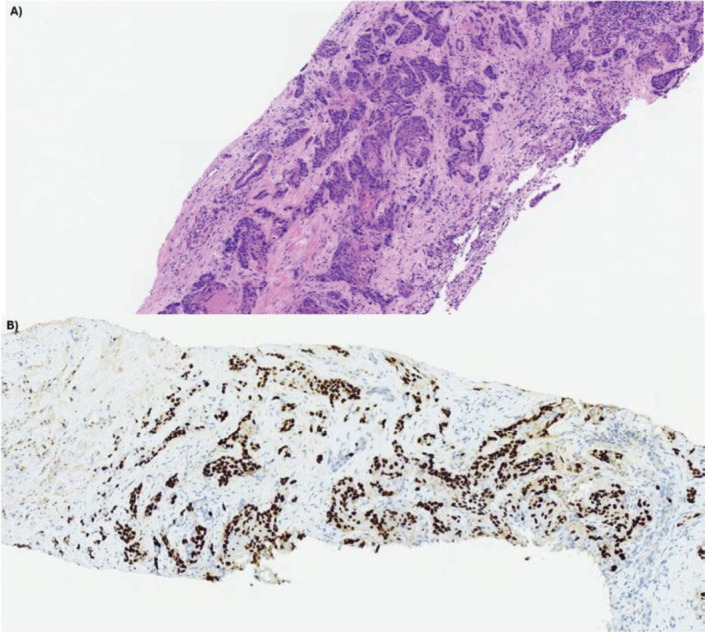
Anatomopathological and immunohistochemical analysis of liver biopsy at the time of recurrence. (a): Liver biopsy sample dated 05/16/2022, stained with hematoxylin and eosin (H&E), revealing parenchymal infiltration by irregular squamous cell aggregates. (b): Immunohistochemical analysis of the same sample demonstrates strong and diffuse positivity for high molecular weight cytokeratin 34Beta (Ventana, clone 34Be12).

**Figure 3.**
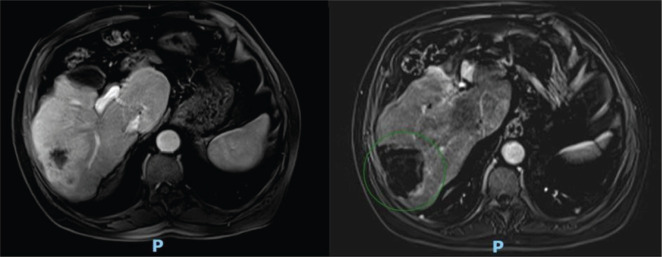
Clinical response of a right lobe liver lesion after microwave ablation. On the left, an axial pos-arterial contrast magnetic resonance imaging (MRI) of the upper abdomen with Primovist, from 05/16/2022, showing a hepatic mass in the posterior sector of the right lobe measuring 6.3 × 3.7 cm, with a large area of enhancement. On the right, a axial, post-arterial contrast subtraction MRI from 07/19/2022, with resolution of the enhancement area.

After 21 months of follow-up, in August 2021, whole-body FDG PET/CT detected suspicious lesions at the left iliac bone wing level, sternal region and a nodule in segment VII of the liver, as illustrated in Figure 1. A biopsy of the left iliac bone confirmed metastatic squamous cell carcinoma, and immunohistochemistry indicated a combined proportional score < 1, considered negative. First-line systemic therapy was initiated with docetaxel at 75 mg/m² and cisplatin at 75 mg/m² on day 1 and cetuximab on days 1, 8 and 15 (400 mg/m² on day 1 of cycle 1 and 250 mg/m² weekly thereafter) every 21 days for four cycles (TPEx protocol), with poor tolerance, marked by hospitalisation due to severe cutaneous reactions and pneumonia, resulting in discontinuation after 3 months of treatment and resumption of anti-EGFR therapy alone. After 10 months, in June 2022, the patient developed a new isolated liver progressive disease (PD) (Figure 2). Abdominal magnetic resonance imaging was performed (Figure 3). As a surgical procedure was deemed unsuitable due to prior liver cirrhosis and clinically significant portal hypertension, coupled with exclusive hepatic involvement in an asymptomatic patient, local therapy with microwave ablation guided by tomography was recommended and cetuximab treatment was continued.

Two months later, the patient experienced worsening pain, and imaging identified PD in the left iliac bone and a pathological fracture in the L4 vertebra. Stereotactic body radiation therapy (SBRT) was administered for both sites, with a total dose of 27 Gray (Gy) delivered in 3 fractions of 9Gy each. Concurrently, systemic therapy with nivolumab was started at a dose of 3 mg/kg every 2 weeks. The patient developed a new bone PD in the sternal region with a pathological fracture after 2 months. Again, SBRT was performed at the same schedule, and checkpoint inhibitor therapy was continued. After 3 months of treatment onset, the dose was adjusted to 480 mg every 4 weeks for patient convenience. At the last follow-up visit on August 19, 2024, after 24 months of initiating nivolumab, the patient remained asymptomatic, with hypothyroidism as the only immune-mediated adverse reaction and excellent functionality. Follow-up CT scans demonstrate a complete and sustained response without evidence of disease activity.

## Discussion

In this case report, we describe an oligometastatic laryngeal carcinoma with metastases to bone and liver that responded remarkably to a combination of local therapies, including microwave ablation (MWA) and SBRT, along with immunotherapy. Although formal guidelines for managing oligometastasis are not established, leading societies recommend polychemotherapy with or without an anti-EGFR agent as a standard treatment for PD-L1 negative patients. The notable success of this combined treatment approach, showing a complete and lasting response with excellent tolerance, suggests a promising synergy between local therapies and immunotherapy. To the best of our knowledge, this case is one of the pioneering reports of this treatment strategy for laryngeal carcinoma patients.

The implementation of local therapies in head and neck squamous cell carcinoma (HNSCC) is increasingly pivotal in oncologic practice, as evidenced by the success observed in Human papillomavirus related oropharyngeal cancer, where radiotherapy and surgery undergo assessment for de-intensification in selected patients [6]. The medical literature reveals a modest amount of data when exploring the synergy between immunotherapy and local therapies. McBride *et al* [7] evaluated the interaction between anti-PD-1 therapy and radiotherapy through the abscopal effect, resulting in a negative outcome for the primary endpoint of the overall response rate [7]. Data on ablative techniques are even more limited, with no reports available for their use in laryngeal cancer. However, they have occasionally been employed for local control of oropharyngeal and thyroid cancer recurrences.

A key observation is the clinical activity of introducing immunotherapy when standard chemotherapy yields suboptimal outcomes, as reaffirmed by the KEYNOTE-048 study for recurrent and metastatic HNSCC [4]. These findings are further supported by the CheckMate141 study, which also included PD-L1 negative patients and improved overall survival, reduction of symptoms and maintenance of functional quality of life with nivolumab-based immunotherapy. Our patient's combination of local therapy and immunotherapy resulted in a sustained clinical response, with 18 months of treatment without disease progression to date. This outcome significantly exceeded the 2 months of progression-free survival typically expected with immunotherapy alone in similar scenarios, as observed in the CheckMate-141 trial [5].

Microwave ablation is a minimally invasive thermal technique increasingly used for treating various tumours, including those in the liver. Recent data from the MAVERRIC trial, supported by the findings of the COLLISION trial presentation, highlight that MWA is non-inferior to hepatic surgical resection in terms of oncological outcomes for metastatic colorectal carcinoma, particularly for small and few lesions. Moreover, MWA offers significant advantages, including reduced post-procedural morbidity and mortality, shorter hospital stays, lower incremental costs and additional options for retreatment in cases of liver metastasis recurrence [8,9].

The choice of microwave ablation for the hepatic lesion in our patient was influenced by clear surgical contraindications due to hepatic cirrhosis, with the liver being the only site of active distant disease and the fact that the patient was asymptomatic and had excellent clinical performance. After achieving successful local control, a notable and sustained clinical response to nivolumab was observed despite negative PD-L1 testing. These findings suggest that the combined effects of immunotherapy and local therapies might generate a supportive environment for immunotherapy, even in patients with adverse predictive biomarkers.

The primary limitation of our case report is attributed to the inability to directly or indirectly measure antigenic release and other parameters of response to immunotherapy, such as evaluating lymphocytic clonality and performance through the neutrophil-to-lymphocyte ratio, as previously reported by Takeda *et al* [10] during the use of nivolumab in a preoperative context for lung cancer. In clinical application, the foremost challenge lies in identifying the most suitable candidates and addressing the absence of reliable biomarkers.

## Conclusion

Combining local therapies and immunotherapy appears to be a promising approach for managing oligometastasis in laryngeal cancer. Despite the theoretical basis for this approach, larger studies are required to validate its impact on oncological outcomes and quality of life. Challenges related to patient selection, biomarker relevance and response evaluation can affect how the results of immunotherapy are interpreted. Polychemotherapy, with or without anti-EGFR agents, continues to be the standard treatment for this patient profile. The relevance of this case is ultimately emphasised as it prompts discussion about the management strategies for oligometastatic HNSCC, especially under the challenging circumstances of a PD-L1negative patient.

## Conflicts of interest

João Felipe Feldmann, João Henrique Feldmann, Cassio Murilo Hidalgo-Filho, Amanda Acioli de Almeida Robatto, Breno Jeha Araujo, Publio Cesar Cavalcante Viana: Declare no conflicts of interest.

## Gilberto de Castro Junior:

Honoraria: AstraZeneca, Pfizer, Merck Sharp & Dohme, Bristol Myers Squibb, Novartis, Roche, Amgen, Janssen, Merck Serono, Lilly, Takeda, Daiichi Sankyo/UCB Japan.

Consulting or Advisory Role: Boehringer Ingelheim, Pfizer, Bayer, Roche, Merck Sharp & Dohme, Bristol Myers Squibb, AstraZeneca, Yuhan, Merck Serono, Janssen, Libbs, Sanofi, Novartis, Lilly, Takeda, Daiichi Sankyo/UCB Japan.

Speakers' Bureau: AstraZeneca, Bayer, Novartis, Roche, Merck Serono, Bristol Myers Squibb, Merck Sharp & Dohme, Boehringer Ingelheim, Pfizer, Janssen, Amgen, Takeda.

Travel, Accommodations, Expenses: Merck Sharp & Dohme, Novartis, Pfizer, Roche, AstraZeneca, Boehringer Ingelheim, Bayer, Bristol Myers Squibb, Merck Serono, Daiichi Sankyo/UCB Japan.

## Funding

No funding was received for this study.

## Ethical statement

Ethical approval for this study obtained from Hospital Sirio-Libanês Ethics Committee, (CAEE 80867724.2.0000.5461) in July 16th 2024.

## Patient’s consent

Patient’s informed consent was obtained for this case report.

## Author contributions

João Felipe Feldmann, João Henrique Feldmann, Cassio Murilo Hidalgo-Filho:

Conceptualisation, methodology, project administration, supervision, analysis and interpretation of data, visualisation, formal analysis, writing original draft, writing review and editing, final approval of the version to be submitted.

Amanda Acioli de Almeida Robatto, Breno Jeha Araujo, Publio Cesar Cavalcante Viana: supervision, analysis and interpretation of data, visualisation, writing review and editing, final approval of the version to be submitted.

Gilberto de Castro Junior: Conceptualisation, methodology, project administration, supervision, visualisation, writing review, editing and final approval of the version to be submitted.

## Data availability statement

The data that support the findings of this case report are available from the corresponding author upon reasonable request. Due to privacy and ethical concerns, the data are not publicly available. Access to the data is subject to the approval of the institution's ethical review board and may require additional patient consent.
